# Long-Term Effects of Acceptance and Rejection by Parents and Peers on Educational Attainment: A Study from Pre-Adolescence to Early Adulthood

**DOI:** 10.1007/s10964-021-01506-z

**Published:** 2021-10-05

**Authors:** Sofie J. Lorijn, Maaike C. Engels, Mark Huisman, René Veenstra

**Affiliations:** Department of Sociology, University of Groningen, Groningen, the Netherlands

**Keywords:** Educational attainment, Acceptance, Rejection, Parents, Peers, Longitudinal

## Abstract

Acceptance and rejection by parents and peers play an important role in pre-adolescents’ educational outcomes. Prior research focused on either parents or peers, did not encompass effects into adulthood, or considered either acceptance or rejection. This study investigated the relation between parental and peer acceptance and rejection, and their interplay, in pre-adolescence and educational attainment in early adulthood. A sample of 2229 pre-adolescents (*M*_age_ T1 = 11.11, SD = 0.56; 50.7% girls) was followed to early adulthood (*M*_age_ T5 = 22.29, SD = 0.65). Ordinal logistic regression showed that pre-adolescents’ perceived parental acceptance was positively related to educational attainment in early adulthood, whereas peer rejection was negatively related, even when WISC score and socioeconomic status were considered. No interaction effects were found, revealing no “dual-hit effect” of being rejected by parents and peers, no “dual-miss effect” of being accepted by parents and peers, and no effects of acceptance in one context (i.e., parents or peers) buffering the negative effect of rejection in the other context. The findings underscore unique and long-term links of parental acceptance and peer rejection with early adults’ educational attainment, underlining the importance of not only peers but also parents in adolescence. These insights can be used in promoting long-term educational outcomes through relationships with parents and peers.

## Introduction

Parents and peers are the most important social figures for pre-adolescents (Hartup, 1979). Feeling accepted by parents reflects relationships in which pre-adolescents perceive their parents to be warm and loving, whereas feeling rejected refers to pre-adolescents perceiving their parents to treat them coldly. Being accepted by peers means being liked by classmates or having friends in class, whereas being rejected by peers refers to being disliked in class. More acceptance from parents and peers is positively related to higher academic achievement and attaining higher levels of education (e.g., Chen, 2017; Wang & Eccles, 2012; Wentzel et al., 2021). In contrast, more rejection from parents and peers is associated with lower academic achievement and educational attainment (e.g., Ali, 2011; Cillessen & van den Berg, 2012). To date, however, most studies on the relation between acceptance and rejection by parents and peers and educational attainment have three main shortcomings. First, prior research on the effects of acceptance and rejection on educational outcomes considered parents and peers separately. Whereas parents are considered the main attachment figures, relationships with peers gain importance in adolescence, greatly impacting adolescents’ development. However, a sole focus on peers may misprize indirect parental effects through peers, not revealing to what extent peers and parents uniquely impact adolescents’ development and to what extent parents remain of importance in adolescence. In addition, investigating parents and peers separately leaves it unclear how relationships with parents and peers jointly impact educational attainment. For instance, it is unknown if being accepted by peers can buffer the negative effects of being rejected by parents on long-term educational attainment. Second, most studies on this relation do not encompass effects into early adulthood. Consequently, little is known about the long-term effects of these social relationships on educational attainment. Focusing on educational outcomes in early adulthood is particularly interesting, because at this stage of life, young adults generally complete their studies and enter the job market. Investigating early adults’ educational attainment thus reflects the long-term goal of education to stimulate individuals to complete their highest possible level of education. Third, most previous studies considered either acceptance or rejection. However, acceptance and rejection are not two sides of the same coin, as the absence of being accepted is not the same as being rejected and vice versa (Markus et al., 2003). Consequently, acceptance and rejection may have unique effects on educational outcomes, stressing the need to investigate both acceptance and rejection (Engels et al., 2019). This study aimed to contribute to the literature by (1) taking the contexts of parents and peers into account simultaneously, (2) examining educational attainment in early adulthood, and (3) considering both acceptance and rejection. These insights can be used in promoting long-term educational outcomes through relationships with parents and peers.

### Acceptance and Rejection by Parents and Peers

Perceived parental acceptance and rejection greatly impact a child’s development. Following Bowlby, this can mainly be explained by the attachment relationship: the deep and enduring emotional bond between parents and child (Bowlby, 1973). Acceptance and rejection constitute the most important factors in attachment relationships. Perceived acceptance reflects responsive, predictable, and sensitive responses of parents to their child’s needs, leading to secure attachments. Rejection refers to insensitive and arbitrary responses, resulting in insecure attachments. Secure attachments foster the child’s self-worth and trust in others and provide a safe base from which the child can explore and develop. Although attachment theorists stress the importance of early attachment, parental attachment remains important in adolescence. In adolescence, perceived parental acceptance encompasses parents being loving and accepting, showing interest in school work, encouraging when their child is doing well and comforting when their child is upset. Parental rejection in adolescence may include being treated harshly and coldly, being disproportionally punished for bad behavior, being blamed, or getting treated differently than siblings. Similar to processes in infancy and childhood, parental acceptance fosters cognitive development in adolescence (Steinberg, 2001).

In addition to parents, peers are an important source of acceptance and rejection. Especially during adolescence, peers become more salient (Veenstra & Laninga‐Wijnen, 2021). Contact with peers increases in adolescence, which can be explained by adolescents’ need for autonomy and independence from their parents. As such, establishing and maintaining peer relationships is an important developmental task in adolescence (Bagwell & Bukowski, 2018). Peer relationships are unique in that they are more equal, less controlling, and less judgmental than relationships with parents and other adults. Whereas parents are “supposed” to love their children, this is not the case with peers (Giordano, 1995). Therefore, although peers are generally not seen as attachment figures, they are attractive social figures for gaining acceptance and realizing a sense of belonging (Baumeister & Leary, 1995). In particular, friends are important for pre-adolescents’ belonging, as friendships are the closest type of voluntary and reciprocal social relationships (Furman & Rose, 2015).

Classmates are the most relevant peers when considering the effects of peer acceptance and rejection on educational attainment. In the context of the classroom, acceptance reflects the extent to which the adolescent has reciprocal in-class friendships or the adolescent is liked by classmates. In practice, peer acceptance may include having friends to hang out with during breaks, being encouraged by peers for doing good work, receiving academic help, and being comforted when feeling down. Rejection refers to the extent to which adolescents are disliked by classmates (Veenstra et al., 2018). Being rejected by classmates may imply being excluded, receiving negative reactions to behavior, and not having friends to hang out with or reach out to for emotional support.

### Parental Acceptance and Rejection and Educational Attainment

Many studies show the importance of perceived parental acceptance and rejection for a child’s socio-emotional development (Khaleque & Rohner, 2002). However, relatively little is known about the impact of perceived parental acceptance and rejection on academic development. In addition, most studies focused on childhood, failing to investigate the continued importance of parents in adolescence. The few studies on this topic found that pre-adolescents more often obtain higher grades when perceiving more parental acceptance (Kim et al., 2002). An important mechanism through which educational attainment is fostered by parental acceptance is self-worth (Chen, 2017). Perceived parental acceptance contributes to adolescents’ self-image of being competent to learn, which provides the confidence to explore academic contexts. This in turn fosters adolescents’ academic motivation and engagement (Wang & Eccles, 2012). Moreover, perceived parental acceptance fulfills the need to belong, which is viewed as a precondition for intrinsic motivation (Ryan & Deci, 2000). In addition, perceived parental acceptance is linked to fewer internalizing and externalizing problems (Madigan et al., 2016), which can undermine academic achievement. For example, both internalizing and externalizing problems can hamper academic achievement by causing stress and a lack of concentration in class.

Even less is known about the effects of perceived parental rejection on educational attainment, but a negative relation has been found between perceived parental rejection and academic achievement (Ali, 2011). Perceiving rejection from parents may generate insecurities about learning and create negative self-fulfilling prophecies. Moreover, perceived parental rejection is associated with lower achievement motivation (Ralte & Fente, 2018), psychological maladjustment (Khaleque & Rohner, 2002), and emotional instability (Mendo-Lázaro et al., 2019), which may hamper academic achievement.

Perceived parental acceptance and rejection may have long-term consequences for academic achievement and later educational attainment. Parental acceptance in infancy was found to be related to reaching higher levels of education and better occupational functioning in adulthood (Raby et al., 2015). Given the rather stable nature of parental acceptance over time, this finding is not surprising (Fraley, 2002). However, besides indirect effects (through later parental acceptance), direct effects of parental acceptance in infancy on educational attainment in adulthood were found (Englund et al., 2011). This underlines the importance of investigating the long-term effects of parental acceptance and rejection. Based on previous studies, it was expected that perceived parental acceptance in pre-adolescence would be positively related to educational attainment in early adulthood (Hypothesis 1). Long-term studies of perceived parental rejection are lacking. Cross-sectional studies show the negative effect of parental rejection on educational attainment in (early) adulthood (Ralte & Fente, 2018). From a theoretical perspective, parental rejection in adolescence may be so harmful that it has long-term negative consequences for educational attainment, even if the relationship between parents and peers improves in later adolescence. Thus, it was expected that perceived parental rejection would be negatively related to educational attainment in early adulthood (Hypothesis 2).

### Peer Acceptance and Rejection and Educational Attainment

Similar to acceptance and rejection by parents, acceptance and rejection by peers affect pre-adolescents’ academic achievement. Receiving more acceptance from peers is linked to obtaining higher grades (Wentzel et al., 2021). Peer acceptance can foster academic achievement in several ways. For instance, peer acceptance has been associated with higher school engagement, school well-being, receiving more academic support, and feeling more comfortable in the classroom, which can promote students’ academic achievement (Kiuru et al., 2020; Wentzel, 2017; Wentzel et al., 2021). For pre-adolescents who are accepted by peers, going to school may be rewarding socially and academically (Ryan & Shin, 2018).

Besides positive effects, peer acceptance may have negative consequences for academic achievement, depending on the behavior of peers. Some peers may approve of problem behaviors and non-compliant school behaviors. For example, having friends with antisocial values has a negative effect on school compliance (Wang & Eccles, 2012). Moreover, popular and liked adolescents were found to have lower levels of behavioral engagement and higher behavioral disaffection than unpopular and disliked adolescents (Engels et al., 2019). Thus, being accepted by friends with non-compliant school behaviors might be a risk for future educational attainment. This is particularly the case for older adolescents who are in secondary education. For pre-adolescents who are in primary education, being accepted by peers generally positively relates to academic achievement (Wentzel et al., 2021).

Being rejected by peers negatively affects academic achievement. Pre-adolescents who are rejected by peers are more likely to skip school, drop out, and obtain lower grades (Cillessen & van den Berg, 2012; Véronneau & Dishion, 2011). In addition, peer rejection is associated with lower school well-being, school interest, and academic self-perception, and higher levels of depressive symptoms, which undermine academic achievement (Ryan & Shin, 2018; Verschueren et al., 2012; Wentzel, 2017; Yang et al., 2020).

Studies of the long-term effects of peer acceptance and rejection are scarce (Veenstra & Laninga‐Wijnen, 2021). Exceptions show evidence for the existence of long-term effects. Pre-adolescents who were accepted by peers reached higher levels of education in early adulthood (Loeb et al., 2020). Pre-adolescents who were rejected by peers showed decreases in academic development, lower educational attainment, lower job competence, and more unemployment in (early) adulthood (Gest et al., 2006; Véronneau et al., 2010). Additionally, peer acceptance and rejection are relatively stable during adolescence (Engels et al., 2019), suggesting that adolescents who experience peer acceptance or rejection in their earlier years are likely to experience similar levels later in their lives. Considering the abovementioned studies, it was expected that pre-adolescent peer acceptance would be positively (Hypothesis 3) and pre-adolescent peer rejection would be negatively (Hypothesis 4) related to educational attainment in early adulthood.

### Parents and Peers: Interrelated Contexts

Relationships with parents and peers differ in several aspects. Parents may play a larger role in mechanisms already at play in infancy, such as fostering feelings of general self-worth, whereas peers may have a larger impact on processes within the classroom, such as in-class academic support and school well-being. Correspondingly, previous studies have shown how parents and peers uniquely affect adolescents’ educational outcomes. For example, peer acceptance had a larger effect on school compliance, whereas parental acceptance had a larger effect on adolescents’ school identification and subjective value of learning (Wang & Eccles, 2012). Moreover, whereas peer acceptance was solely related to social self-concept, parental acceptance uniquely contributed to general self-concept (Verschueren et al., 2012). However, both parents and peers are important sources of acceptance and rejection, and both can provide affection, feelings of belonging, and emotional and academic support. This raises the question to what extent relationships with parents and peers interact when taken into account simultaneously (Sentse et al., 2010).

The interaction between social contexts is based in the socio-ecological systems theory, which argues that adolescents develop in interaction with their surrounding social environment, with the family and the peer group forming the most immediate contexts (Bronfenbrenner, 1979). Consequently, relationships with parents and peers do not act in isolation but are interconnected. Adolescents who perceive themselves to be accepted by parents are more likely to also be accepted by peers, and the same holds for rejection (Gorrese & Ruggieri, 2012). This is in line with Bowlby’s attachment theory, which states that relationships with parents establish patterns for later social relationships, such as with peers (Bowlby, 1973; Mak et al., 2018). Moreover, relationships with peers affect relationships with parents. For instance, being rejected by peers increases maladjustment, which in turn can lead to negative interaction with parents (Kaufman et al., 2020).

Interdependencies between the contexts of parents and peers may lead to several interactions between acceptance and rejection by parents and peers on educational outcomes. First, acceptance and rejection from parents and peers may have positive synergetic effects. This means that the positive effects of acceptance on educational attainment is larger when acceptance is experienced in both contexts (i.e., parents and peers) compared with acceptance in only one context (i.e., parents or peers) (Fass & Tubman, 2002). This synergic effect may occur because these adolescents benefit from a “dual-miss effect”: they do not experience the negative consequences of low acceptance from parents or peers. Besides, parental acceptance may promote peer acceptance and vice versa, creating a positive cycle promoting future educational attainment. Conversely, rejection by parents and peers could establish a “dual-hit effect” (Hazel et al., 2014). This means that the negative effects of rejection on educational attainment are larger when both parents and peers are rejecting, compared with rejection in only one social context (Fass & Tubman, 2002). Although studies on this “dual-hit effect” on educational outcomes are scarce, this double negative effect has been found for delinquency, well-being, and general functioning (Richards et al., 2019; Van der Laan et al., 2010). Considering these findings, it was expected that parental and peer acceptance would reinforce each other in a positive way (“dual-miss effect”; Hypothesis 5a) and that parental and peer rejection would reinforce each other in a negative way (“dual-hit effect”; Hypothesis 5b).

Second, acceptance and rejection from parents and peers might, to some extent, compensate for each other (Cohen & Wills, 1985). For instance, the negative effects of parental rejection can be smaller for adolescents who are accepted by peers. Although this “buffer effect” was found for victimization, internalizing problems, externalizing problems, and self-esteem (Birkeland et al., 2014; Nakamoto & Schwartz, 2010; Sentse et al., 2010), there is no prior research on educational outcomes. However, considering that these factors are important mechanisms in facilitating educational attainment, it was expected that peer acceptance would be able to partially buffer for the negative effects of perceived parental rejection on educational attainment (Hypothesis 6). Findings are contradictory when it comes to the question of whether perceived parental acceptance can buffer for the negative effects of peer rejection. Maternal acceptance has been found to buffer for the negative effect of peer rejection on perceived social competence (Rubin et al., 2004) and internalizing problems (Zarra-Nezhad et al., 2019). Moreover, parental acceptance was found to interact with peer victimization (Bowes et al., 2010). Bullied children who were accepted by parents experienced fewer emotional and behavioral problems than bullied children who were not accepted by parents. However, perceived parental acceptance did not buffer the negative effects of peer rejection on internalizing and externalizing problems (Sentse et al., 2010). Therefore, it was explored whether perceived parental acceptance buffered the negative effects of peer rejection on long-term educational attainment.

## Current Study

Prior research on the association between acceptance and rejection by parents and peers and educational outcomes focused on either parents or peers, did not encompass effects into early adulthood, or considered either acceptance or rejection. This study investigated how acceptance and rejection from parents and peers, and their interplay, in pre-adolescence are associated with educational attainment in early adulthood. Thereby, this study overcomes several gaps in the literature that limit our knowledge of how pre-adolescents’ long-term educational outcomes can be promoted through relationships with parents and peers. It was expected that perceived parental acceptance and peer acceptance would contribute to educational attainment, whereas perceived parental rejection and peer rejection would hamper it. Furthermore, the interplay between parental and peer acceptance and rejection was examined, expecting a “dual-miss effect,” a “dual-hit effect,” and buffering effects.

## Methods

### Procedure and Sample

Data stem from the Tracking Adolescents’ Individual Lives Survey (TRAILS). TRAILS was designed to chart and explain the development of mental health and social development from pre-adolescence into adulthood (De Winter et al., 2005). The target sample consisted of pre-adolescents aged 11 from five municipalities in the north of the Netherlands. All primary schools in these municipalities were approached for participation in the study, encompassing 135 schools with 3483 pre-adolescents. Thirteen schools refused to participate (90.4% response rate of schools), excluding 338 of the 3483 pre-adolescents. When the school was willing to participate, children and parents were informed about the study and invited to participate. Of all 3145 pre-adolescents approached, 210 (6.7%) were excluded because of incapability or language problems; 76.0% of the remaining 2935 pre-adolescents and their parents responded to the invitation and agreed to participate, resulting in a sample size of 2229. Participants had a mean age of 11.1 at T1, 50.7% of the participants were girls, 89.4% of the participants had a western background, and 74.8% of the participants came from moderately to highly urbanized areas. Detailed descriptions of TRAILS can be found elsewhere (Oldehinkel et al., 2015). Data from the first and fifth waves (*M*_age_ = 22.3 years) of TRAILS were used.

### Missing Data

There were two types of missing data in this study. First, there was missing data by design due to the use of a peer subsample. This subsample of 1065 pre-adolescents participated in a complementary data collection of peer nominations at T1 (see also Veenstra et al., 2005). The assessments of the peer nominations lasted for about 15 min and took place during regular lessons. After brief instructions in which a TRAILS staff member emphasized that information would be kept confidential, the participants received the questionnaire with the names of the classmates listed. Peer nominations were only assessed in classrooms with at least ten TRAILS respondents. As a result, this peer subsample is more selective. Pre-adolescents in special education (5.6% of the sample) and small schools (6.4%), and those who repeated a grade (16.9%) or skipped a grade (2.2%) were not part of the subsample. Second, there was missing data because of attrition. This study includes data from the first assessment wave (T1), when the respondents were approximately 11 years old, and the fifth assessment wave (T5), when they were approximately 22 years old. Of the 2229 pre-adolescents at T1, 1430 (64.2%) reported their educational attainment at T5.

Missing data were handled by multiple imputations using Multivariate Imputation by Chained Equations (MICE) in R (Van Buuren, 2018). The data were imputed 50 times, resulting in 50 completed datasets (*N* = 2229) that were analyzed separately, after which results were pooled to obtain the final results (for both descriptive and confirmatory analysis). Thus, the results are shown for a sample size of 2229. For the logistic regressions in Tables 3 and 5, the fraction of missing information (FMI) is provided. The FMI shows the proportion of the variation in the estimate due to missing data (adjusted for the number of imputations), and is an indicator of the severity of the problem caused by missing data (Van Buuren, 2018). Values up to 0.2 show “modest,” 0.3 “moderately large,” and 0.5 “high” fractions of missing information. High values indicate that the statistical inference is dependent on the missing data treatment procedure. Appendix A gives more information on the imputation procedure and choices, and includes descriptive statistics for the incomplete sample and the multiply imputed sample in Table 4.

### Measures

#### Educational attainment

Educational attainment was assessed at T5, combining the highest completed degree and, if applicable, the level of current education. The level of current education was assigned if this was higher than the level of obtained education. The Dutch educational system is hierarchical in nature and can be categorized into four levels: lower secondary vocational education (1), higher secondary vocational education (2), University of Applied Sciences (3), and university (4). Missing information on educational attainment was filled in using previous assessments and the event history calendar at T5 (Schmengler et al., 2021).

#### Perceived parental acceptance and rejection

Perceived parental acceptance and parental rejection were assessed at T1 using the shortened EMBU-C scale (Markus et al., 2003). This scale measures the child’s perceptions of acceptance and rejection from their mother and father. The scale includes 18 items for acceptance (internal consistency *α* = 0.91 for fathers and *α* = 0.90 for mothers) and 17 items for rejection (internal consistency *α* = 0.85 for fathers and *α* = 0.82 for mothers). Prior research found the test-retest stability of this scale over a two-month period to be satisfactory (*r* = 0.78 or higher; Muris et al., 2003) and the validity to be good (Markus et al., 2003).

Parental acceptance (Emotional Warmth subscale) was characterized by children’s perceptions of parents’ unconditional love, affection, praising of approved behavior, showing interest, and supportive behavior. Sample items are “Does your mother/father show you that they love you?” and “Do you have the feeling that your mother/father likes being with you?”. Parental rejection is characterized by children’s perceptions of parents’ hostility, punishment, derogation, and blaming. Sample items are “Does your mother/father sometimes punish you even though you haven’t done anything wrong?” and “Is your mother/father sometimes harsh and unkind to you?”. Respondents could rate the EMBU-C as 1 = no, never, 2 = yes, sometimes, 3 = yes, often, and 4 = yes, almost always. The answers for both parents were highly correlated (*r* = 0.81 for acceptance and 0.73 for rejection) and therefore averaged.

#### Peer acceptance and rejection

Peer acceptance and peer rejection were assessed at T1 using classroom-based peer nominations. Pre-adolescents were asked which classmates were their best friends (acceptance) and which classmates they disliked (rejection), for both of which they could nominate an unlimited number of classmates. Nominations received for being best friends and for being disliked were divided by the total number of classmates. This way, the scores were transformed into proportions, taking differences in class size into consideration. Scores for peer acceptance and peer rejection ranged from 0 to 1, with higher scores indicating more acceptance or rejection. This procedure is a commonly used and reliable way to treat peer nominations (Veenstra et al., 2018).

#### Gender

Gender (0 = girl; 1 = boy) was measured at T1 and included to control for possible gender differences in educational attainment.

#### WISC score

The vocabulary and block design subtests of the Wechsler Intelligence Scales for Children-Revised (WISC-R; Wechsler, 1974) were used as an approximation of intelligence (Silverstein, 1975), measured at T1. These two subtests were chosen based on their high correlation (*r* = 0.90) with the complete WISC-R in prior research (Silverstein, 1975).

#### Socioeconomic status

Socioeconomic status combined mothers’ and fathers’ educational levels and occupational levels, and household income. Missing values, for example, for single parent households or unemployed parents, hardly affected this scale. Therefore, socioeconomic status was calculated per family.

### Analysis

First, descriptive statistics were explored for all study variables separately for level of education and the correlations between the predictors were calculated. Second, two ordinal logistic regression models were applied to assess whether acceptance and rejection from parents and peers at 11 years of age, and their interplay, were associated with educational attainment 11 years later. The models were estimated using the GLM procedure in SPSS. The first model included acceptance and rejection by parents and peers as predictors of educational attainment. In the second model, four interaction effects were added: namely, the interactions between parental and peer acceptance; parental and peer rejection; parental acceptance and peer rejection; and parental rejection and peer acceptance. These interactions were added to test for enhancing and buffering effects. All models controlled for adolescents’ gender, WISC score, and socioeconomic status at T1. To allow interactions in the model, the predictors were centered to *M* = 0. Results shown are for the pooled estimates of the 50 multiply imputed datasets.

Model fit was assessed using Akaike information criterion (AIC) and McFadden’s and Nagelkerke’s pseudo-*R* tests. Lower values of AIC indicate a better model fit. McFadden’s *R*-squared is a pseudo-*R* test with values closer to zero indicating that the model has low predictive power, and values between 0.2 and 0.4 showing a very good model fit. Nagelkerke indicates the goodness of fit for a model on a scale from 0 to 1, thus rules of thumb for regular *R*-squared apply.

All the VIF scores were below the critical value of 4, indicating no multicollinearity. If, for each explanatory variable in the model, the effect is the same for each pair of outcome groups, one estimated effect can be provided. This assumption of proportional odds was inspected using cumulative binary logistic regressions (Long & Freese, 2006). Table 5 in Appendix B shows the cumulative binary logistic regressions. Inspection per variable shows that the assumption was met for most of the variables that were significant in the main analyses. Only for peer rejection was the negative effect larger when the highest level of education was compared with the three lower levels.

## Results

### Descriptive Statistics

Table 1 presents the descriptive statistics of the study variables by attained educational level. Most early adults (*N* = 1248, 56%) reached the University of Applied Sciences level or higher, whereas 19% of early adults did not achieve a basic qualification, referring to lower vocational education. In pre-adolescence, participants overall perceived and received more acceptance than rejection from parents as well as from peers. Table 1 shows that socioeconomic status and WISC score varied strongly across educational levels: the higher the level of SES and intelligence at T1, the higher the educational attainment at T5. Perceived parental and peer acceptance and rejection also varied across educational levels, with greater differences for perceived parental acceptance and peer rejection. Girls generally reached higher levels of education in early adulthood than boys (*F*(3, 320.93) = 3.63, *p* = 0.013).

**Table 1:** Descriptive statistics for study variables by educational attainment (*N* = 2229)

Variables	Lower vocational education (*n* = 423.3)	Higher vocational education (*n* = 557.2)	University of Applied Sciences (*n* = 830)	University (*n* = 418.5)			Differences	
	Mean (SD)	Mean (SD)	Mean (SD)	Mean (SD)	Min	Max	*F*	*p*
WISC score	84.82 (13.05)	92.56 (12.24)	100.25 (12.60)	109.66 (12.46)	45	149	231.05	<0.001
Socioeconomic status	–0.67 (0.67)	–0.29 (0.68)	0.10 (0.70)	0.59 (0.69)	–1.94	1.73	206.98	<0.001
Parental acceptance	3.04 (0.57)	3.16 (0.48)	3.26 (0.48)	3.37 (0.42)	1.17	4.00	28.37	<0.001
Parental rejection	1.51 (0.37)	1.50 (0.32)	1.48 (0.30)	1.42 (0.25)	1.00	3.47	6.64	<0.001
Peer acceptance	0.26 (0.15)	0.28 (0.16)	0.29 (0.16)	0.30 (0.15)	0.00	0.80	2.94	0.037
Peer rejection	0.18 (0.16)	0.14 (0.14)	0.13 (0.13)	0.10 (0.11)	0.00	0.85	10.53	<0.001

	Frequencies (%)							
Gender	42.6% girls	53.6% girls	50.8% girls	55.2% girls			3.63	0.013
	57.5% boys	46.4% boys	49.2% boys	44.8% boys				

Table 2 shows the correlations between the predictors in the model. Pre-adolescents from higher socioeconomic backgrounds scored higher on the WISC (*r* = 0.39, *p* < 0.001), perceived more acceptance from parents (*r* = 0.15, *p* = 0.001) and were more accepted by peers (*r* = 0.08, *p* = 0.01), and perceived less rejection from parents (*r* = –0.05, *p* = 0.03) and were less rejected by peers (*r* = –0.15, *p* < 0.001). Pre-adolescents who perceived more parental acceptance overall perceived less parental rejection (*r* = –0.32, *p* < 0.001), were less rejected by peers (*r* = –0.09, *p* = 0.01), and more accepted by peers (*r* = 0.11, *p* < 0.001). Perceived parental rejection was related to lower peer acceptance (*r* = –0.16, *p* < 0.001) and higher peer rejection (*r* = 0.14, *p* < 0.001). Pre-adolescents who were more accepted by peers, were less rejected by peers (*r* = –0.38, *p* < 0.001).

**Table 2:** Correlations for predictors and control variables (*N* = 2229)

Variables	1	2	3	4	5	6	7
1. Gender	–						
2. WISC score	–0.06**	–					
3. Socioeconomic status	–0.03	0.39***	–				
4. Parental acceptance T1	–0.10***	0.12***	0.15***	–			
5. Parental rejection T1	0.11***	0.01	–0.05*	–0.32***	–		
6. Peer acceptance T1	–0.07*	0.01	0.08**	0.11***	–0.16***	–	
7. Peer rejection T1	0.20***	–0.09**	–0.15***	–0.09**	0.14***	–0.38***	–

*p ≤ 0.05, **p ≤ 0.01, ***p ≤ 0.001

### Ordinal Logistic Regression Analysis

Ordinal logistic regression models were computed to test whether acceptance and rejection from parents and peers at 11 years, and their interplay, were related to educational attainment 11 years later. First, the main effects were tested in Model 1. Table 3 shows that perceived parental acceptance in pre-adolescence was significantly related to educational attainment at age 22, with pre-adolescents perceiving more parental acceptance being more likely to attain a higher level of education (OR = 1.62, *p* < 0.001). Specifically, when all other characteristics were held constant, each unit increase in perceived parental acceptance resulted in an odds of attaining higher levels of educations that was 1.62 times larger. This result is consistent with the first hypothesis. In contrast, perceived parental rejection was not significantly associated with educational attainment (OR = 0.73, *p* = 0.086); thus, the second hypothesis was not supported. Peer acceptance was not found to change the odds of educational attainment in early adulthood (OR = 1.19, *p* = 0.716), contrary to the third hypothesis, but peer rejection was significantly associated with attaining a lower level of education ten years later (OR = 0.26, *p* = 0.017). This is in line with the fourth hypothesis.

**Table 3:** Ordinal logistic regression on educational attainment (*N* = 2229)

	Model 1			Model 2		
	*B* (SE)	OR	FMI	*B* (SE)	OR	FMI
Gender	–0.24 (0.10)*	0.79	0.32	–0.24 (0.10)*	1.27	0.32
WISC score	0.07 (0.00)***	1.07	0.26	0.07 (0.00)***	1.07	0.26
Socioeconomic status	1.06 (0.07)***	2.89	0.29	1.06 (0.07)***	2.89	0.29
Parental acceptance	0.48 (0.11)***	1.62	0.30	0.48 (0.11)***	1.62	0.29
Parental rejection	–0.31 (0.18)	0.73	0.38	–0.30 (0.18)	0.74	0.34
Peer acceptance	0.17 (0.47)	1.19	0.64	0.20 (0.49)	1.22	0.65
Peer rejection	–1.36 (0.56)*	0.26	0.65	–1.39 (0.57)*	0.25	0.65
Parental × Peer acceptance				–0.20 (0.88)	0.82	0.51
Parental × Peer rejection				1.18 (1.38)	3.25	0.48
Parental acceptance × Peer rejection				0.43 (1.04)	1.54	0.54
Parental rejection × Peer acceptance				0.88 (1.36)	2.41	0.46
AIC	4742.91			4744.70		
McFadden’s pseudo-*R*^2^	21.16%			21.11%		
Nagelkerke pseudo-*R*^2^	46.53%			46.46%		

*p ≤ 0.05, ***p ≤ 0.001

Second, the interaction effects were added in Model 2 to examine the interplay of acceptance and rejection by both parents and peers. Table 3 shows that no significant interactions were found. There was no interaction between perceived parental acceptance and peer acceptance (OR = 0.82, *p* = 0.821), thus no enhancing positive effect when pre-adolescents were accepted by both parents and peers, contrary to H5a. No “dual-hit effect” was found between perceived parental rejection and peer rejection (OR = 3.25, *p* = 0.396); thus, H5b was not supported. There were no interaction effects between perceived parental acceptance and peer rejection (OR = 1.54, *p* = 0.682), or between perceived parental rejection and peer acceptance (OR = 2.41, *p* = 0.521). This means no buffering effects were found, contrary to H6. In addition, the model fit did not improve after the interaction effects were added, with no change to McFadden’s and Nagelkerke pseudo-*R*^2^, and the AIC increased from 4742.9 to 4744.7.

## Discussion

Researchers have stressed the need to integrate the effects of parents and peers (Rubin et al., 2011) and to examine the long-term effects of peer relationships (Veenstra & Laninga‐Wijnen, 2021). However, prior studies on the associations between parental and peer acceptance and rejection and educational outcomes focused on either parents or peers, did not encompass effects into adulthood, or considered either acceptance or rejection. The aim of this study was to examine whether acceptance and rejection from parents and peers, and their interplay, in pre-adolescence is associated with educational attainment 10 years later. This gives insight into whether positive and negative relationships with parents and peers are associated with the level of education that individuals reach. The results show the unique contributions of relationships with parents and with peers in promoting long-term educational attainment. Specifically, pre-adolescents’ perceived parental acceptance was positively, and rejection by peers was negatively related to educational attainment in early adulthood. Only unique effects were found; there were no “dual-hit effects,” “dual-miss effects,” or buffer effects. The results stem from a representative sample of 2229 pre-adolescents from the north of the Netherlands; the theoretical base of the hypotheses implies that the results may be informative for other Western countries.

Regarding parents, the results revealed that pre-adolescents who perceived more parental acceptance attained higher levels of education in early adulthood, even when WISC score and socioeconomic status were considered. In line with previous studies, this finding points to the long-lasting effects of perceived parental acceptance in pre-adolescence (Englund et al., 2011; Raby et al., 2015), as theorized in the attachment theory (Bowlby, 1973). In addition, this finding is in line with previous research arguing that despite adolescence being characterized by gaining independence from parents, perceived parental acceptance remains important (Steinberg, 2001). Independence is not the same as detachment, and pre-adolescents can be accepted by and be independent from their parents at the same time (Ryan & Lynch, 1989). In fact, parental acceptance fosters adolescents’ independence and academic development, similar to processes in infancy (Steinberg, 2001). Although not the focus of this study, the importance of parents is further emphasized by the important role of socioeconomic status, with each unit increase in socioeconomic status in pre-adolescence increasing the odds of attaining a higher level of education by 2.89. Contrary to our expectations in this study, pre-adolescents’ perceived parental rejection was not related to educational attainment 11 years later. In accordance with Bowlby’s attachment theory (1973), being rejected by parents may hamper development in infancy in particular. Despite the relative stability of parental rejection from infancy through adolescence (Waters et al., 2000), the negative consequences of perceived parental rejection may decrease over time, because adolescents become less dependent on their parents. In addition, perceived parental rejection may be an important predictor of some aspects of adolescents’ functioning, but not of educational attainment. For example, a previous TRAILS study found that perceived parental rejection was associated with the development of aggressive and depressive problems in adolescence (Sijtsema et al., 2014).

Peer acceptance in pre-adolescence was not associated with educational attainment in early adulthood, contrary to our expectations in this study. A positive effect of peer acceptance on academic competence may occur especially in relation to high-quality relationships, rather than the number of friendships (Fass & Tubman, 2002). This is in line with the attachment theory, in which the importance of warm and loving attachment relationships is emphasized, rather than merely the presence or quantity of relationships. Furthermore, being accepted by peers might have negative consequences for school outcomes, especially when peers approve of non-compliant school behavior (Ryan & Shin, 2018). Although this negative effect of acceptance for educational outcomes is typically found in secondary education and not in primary education (Wentzel et al., 2021), future studies could investigate whether the effects of peer acceptance on academic achievement in primary education depend on the norms and behaviors of these peers.

Being rejected by peers in pre-adolescence was related to attaining lower levels of education 11 years later, even when WISC scores were taken into account. This confirms that pre-adolescent peer rejection lays a basis for academic achievement and future educational attainment (Gest et al., 2006; Véronneau & Dishion, 2011). These findings suggest that negative peer experiences are more detrimental than a lack of positive peer experiences. Especially considering its stability over time (Engels et al., 2019), peer rejection is an important social factor for educational outcomes. Future research would enrich the literature by examining the (dis)continuation of peer rejection after the transition to secondary education, when students integrate into a new peer environment. Moreover, these findings underline the importance of peers, even when parents are taken into account (Fass & Tubman, 2002). Peers uniquely relate to long-term educational attainment.

The findings imply that relationships with both parents and peers relate to pre-adolescents' long-term educational attainment. No “dual-miss effect,” “dual-hit effect,” or buffer effects were found, contrary to expectations. A previous TRAILS study found that peer acceptance buffered the effects of perceived parental rejection on externalizing and internalizing problems (Sentse et al., 2010). This means that the negative effects of perceived parental rejection were lower for pre-adolescents who were accepted by peers. Whereas the parent and peer contexts are interdependent for externalizing and internalizing problems, parents and peers may uniquely affect pre-adolescents’ cognitive outcomes. In addition, it may be that acceptance and rejection by parents and peers interact in the short term, but that these effects diminish over time. The current study showed that pre-adolescents who perceived more acceptance from parents were generally more accepted by peers, and the same holds for rejection. Relationships with parents often form the foundations for later relationships, such as with peers (Bowlby, 1973). Thus, being accepted in one context and being rejected in the other context might be rare, not leaving room for buffer effects to occur.

The findings of this study underscore the importance of investigating acceptance and rejection separately (Markus et al., 2003). Perceived parental acceptance and peer rejection in pre-adolescence were related to educational attainment in early adulthood. Perceived parental rejection and peer acceptance were not found to be associated with educational attainment. Thus, acceptance and rejection may have differential effects on educational outcomes.

### Limitations and Future Directions

This study has major strengths compared with previous studies. The study made use of a longitudinal design and multiple imputation techniques to handle missing data, the social contexts of parents as well as peers were examined and both acceptance and rejection were considered. However, the findings need to be interpreted in light of the following limitations. First, whereas acceptance and rejection by parents were measured through early adolescents’ perceptions, acceptance and rejection by peers were measured using peer nominations. Using different measures limits the ability to compare acceptance and rejection between different social contexts. Relying on child-reports of parental acceptance and rejection is important because self-reports are more predictive for pre-adolescent outcomes than more objective measures. For instance, the positive effects of parental acceptance on educational outcomes may be absent for pre-adolescents who do not perceive themselves to be accepted by their parents. Accordingly, previous studies determined child-reports to be valid measures of parental acceptance and rejection (Hughes et al., 2005; Jager et al., 2016). No self-reports were available for peer acceptance and rejection, and using peer nominations is recommended (Sentse et al., 2010; Veenstra et al., 2018). Future studies would enrich the literature by using similar measures for acceptance and rejection by parents and peers; this would confirm the unique contributions of parents and peers to long-term educational attainment, while seeking to limit the shortcomings of common method variance (Miljkovitch et al., 2021).

Second, individual characteristics may lead to acceptance and rejection, and educational attainment. The findings revealed that pre-adolescents with higher WISC scores, which is a measure of intelligence, attained higher levels of education in early adulthood. Besides, WISC score was positively correlated with parental acceptance, negatively correlated with peer rejection, and did not correlate with parental rejection and peer acceptance. This may point to a mediation effect of intelligence on educational attainment through social relationships. Similar patterns might occur for other individual characteristics. For instance, adolescent internalizing and externalizing problems can lead to lower parental acceptance (Buist et al., 2004) as well as lower educational attainment in adulthood (Evensen et al., 2016). Distinguishing the effects of acceptance and rejection from those of individual characteristics on educational attainment could be a direction for further research.

Third, related to the second limitation, and because of the nature of this study, no insight was gained into causal relations and underlying mechanisms of the relation between acceptance and rejection in pre-adolescence and educational attainment 11 years later. Although previous research on the bidirectional relation between social relationships and school outcomes has shown that social relationships affect school outcomes (e.g., Engels et al., 2016), school outcomes have also been found to affect social relationships (Mackinnon, 2012). Moreover, future studies could focus on the mechanisms underlying the long-term effects of parental acceptance and peer rejection on educational attainment. Among other factors, self-worth (Chen, 2017), school well-being (Kiuru et al., 2020), and internalizing and externalizing problems (Deighton et al., 2018) could be important mediators. Future longitudinal studies would benefit from including measures in adolescence to gain insight into the processes of the effects of social relationships in pre-adolescence on educational outcomes in adulthood. In addition, future studies would contribute by also taking into account teacher acceptance and rejection and other relevant school characteristics (Sette et al., 2020).

### Practical Implications

The findings of this study point to important practical implications for promoting long-term educational outcomes through relationships with parents and peers. The findings stress the importance of perceived parental acceptance for educational outcomes, thereby underlining that besides school-centric approaches, such as encouraging parents to attend school events or to discuss grades with their children, general parental factors are also important in promoting academic achievement (Malczyk & Lawson, 2019). Providing parents with the tools to create an accepting environment at home supports pre-adolescents to develop academically. In addition to its importance in childhood, parental acceptance remains of significance in adolescence. As in infancy, being accepted by parents creates a safe basis for adolescents to develop (Steinberg, 2001). Thus, programs for improving perceived parental acceptance can be beneficial for promoting adolescents’ long-term educational attainment.

The long-term negative effects of peer rejection show the importance of the social function of education. Feeling safe at school and not being rejected by classmates are important conditions for pre-adolescents’ academic development (Véronneau et al., 2010). Thus, paying attention to preventing pre-adolescents from being rejected by classmates is not done at the expense of time spent on academic development, but rather promotes academic development and future educational attainment (Salmivalli et al., 2012). Gaining insight into adolescents’ social status at school and promoting a favorable peer climate with positive peer relationships can help schools to foster adolescents’ academic achievement and attainment of higher levels of education on the long term.

Considering the unique contributions of parents and peers to long-term educational attainment, programs focused on either social relationships at school (referring to peers) or at home (referring to parents) are promising for promoting long-term educational attainment. For example, anti-bullying interventions may prevent negative peer experiences and foster academic achievement (Salmivalli et al., 2012). Considering the long-term effects of perceived parental acceptance and peer rejection, interventions in pre-adolescence are recommended. Specifically, promoting perceived parental acceptance and preventing peer rejection in pre-adolescence are promising for helping adolescents to reach higher levels of education in the long run.

## Conclusion

Acceptance and rejection by parents and peers are important for pre-adolescents’ academic development and later educational attainment. Whereas prior research focused on either parents or peers, did not encompass effects into adulthood, or considered either acceptance or rejection, this study contributed to adolescent research by examining whether (the interplay of) acceptance and rejection from parents and peers in pre-adolescence is associated with educational attainment in early adulthood. The findings show that parents and peers uniquely contribute to pre-adolescents’ long-term educational attainment. Perceiving more acceptance from parents in pre-adolescence was related to higher educational attainment in early adulthood. Thus, although adolescents become more independent from their parents, positive relationships with parents remain important. Programs to promote educational outcomes may benefit from focusing on more general positive relationships with parents, rather than adopting only school-centric approaches. Peer rejection was related to reaching lower levels of education, showing unique peer effects rather than indirect effects through parents. Preventing peer rejection in classrooms is beneficial for long-term educational outcomes. Pre-adolescents’ long-term educational outcomes can thus be promoted through fostering parental acceptance and preventing peer rejection.

## Appendix A. Information about Multiple Imputation

The TRAILS dataset of 2229 pre-adolescents dropped to 1065 due to the use of a peer subsample and further decreased to 701 because of attrition over 10 years, leading to 68.9% missing data. The 701 participants who were included in the peer subsample and participated in the fifth wave, differed from other TRAILS respondents (*N* = 2229 – 701 = 1528) on several characteristics at T1. The participants included in the subsample were more often girls (59.9%) compared with the participants not included in the subsample ((46.5%) *χ*^2^(1) = 34.43, *p* < 0.001); they had higher socioeconomic backgrounds (*M* = 0.2, SD = 0.74) compared with the participants not included (*M* = –0.16, SD = 0.8) *t*(1447.6) = –10.54, *p* < 0.001); they had higher WISC scores (*M* = 102.35, SD = 13.28) than the participants not included (*M* = 94.81, SD = 15.15), *t*(1537.2) = –11.89, *p* < 0.001); and they achieved better academically (*M* = 3.91, SD = 0.8) compared with the participants not included in the subsample (*M* = 3.48, SD = 0.9), *t*(1327.7) = –10.39, *p* < 0.001). The variables peer acceptance, peer rejection and educational attainment mainly had missing values.

Missing data were handled by using Multivariate Imputation by Chained Equations (MICE) in R (Van Buuren, 2018). MICE creates multiple completed datasets by replacing the missing values with estimated values using the selected method of imputation. Each imputed dataset is analyzed separately, and the analyses results are then pooled to obtain one end result. The pooling is based on Rubin’s rules, taking into account the number of imputations and the increased variances caused by the missing data and the imputation (Van Buuren, 2018).

To optimally impute the data, accurate imputation models were chosen, one for each variable separately. The choice of model is based on two important considerations: (1) the predictor variables to be included in the imputation model, and (2) the nature of the variable to be imputed.

With respect to the first consideration, all imputation models included all variables that were used in data analyses on the imputed data, including interactions. Further, the models contained variables that were related to the non-response or to the variables with missing values (Van Buuren, 2018). The imputation models included available variables related to variables with missing values with a correlation of 0.3 or higher, namely: peer acceptance at T2, peer rejection at T2, academic achievement at T1, school advice at T1 and educational attainment at T2, T3, T4 and T6.

With respect to the second aspect, imputation models were chosen to fit the nature of the variables. That is, categorical variables were imputed using logistic regression models and continuous variables with predicted mean matching (Van Buuren, 2018). Interactions were included as predictors but were not allowed to be imputed themselves, instead they were recomputed using the imputed main variables as soon as these were imputed. Moreover, interactions were not used to impute the two variables included in the interaction.

Generally, it is advised to impute variables using the raw scores rather than the transformed scales. However, the scale of parental acceptance consists of 36 items (18 for mother and 18 for father) and the scale of parental rejection consists of 24 items (12 for mother and 12 for father). Because respondents with missing data on one of these items generally had missing data on most of these items, items within scales cannot predict each other. Therefore, the transformed scale scores (mean scores) were used instead of the original item scores. Furthermore, SES was added as a scale to the imputation model because there were no missing values for SES and because parental occupation is problematic to impute as not being a numerical variable.

For imputation, the variables peer acceptance and peer rejection were transformed using a logit transformation to correct for skewness. The variable peer acceptance consists of the percentage nominations of best friends, ranging from 0 to 1 with a mean of 0.3. The variable peer rejection consists of the percentage nominations of dislikes, ranging from 0 to 1 with a mean of 0.1. Because the logit can only be calculated for scores over 0, the value 0 was transformed into 0.005. After imputation the imputed variables were transformed back to their original values to be used in the data analyses.

Although the default number of imputations is 5, it is advised to impute with a number being approximately equal to the percentage of missing data. However, increasing the number of imputations usually does not change the conclusions from the pooled estimates (Van Buuren, 2018). Therefore, with overall 68.6% missing data we imputed 50 times.

The 50 multiply imputed datasets were imported in SPSS. In SPSS, the logit transformation for the peer variables were reversed. The interaction variables used in the imputations were discarded and new interactions were computed using variables centered on the pooled means. Each imputed dataset was analyzed separately and estimates were pooled by SPSS. Estimates not pooled automatically by SPSS (ANOVA *F* tests, *χ*^2^ tests, SDs, ORs, and model fit indices) were pooled by exporting SPSS results to R and using pooling functions. In the pooling function, degrees of freedom (by default based on the number of imputations) were adjusted for sample size. Table 4 shows the descriptive statistics for the incomplete and the multiply imputed sample. The largest change in descriptive statistics after multiple imputation occurred at the outcome variable. Whereas in the incomplete sample 9.6% of the early adults reached the level of lower vocational education, 24.8% higher vocational education, 41.5% University of Applied Sciences and 24.1% reached university, in the multiply imputed sample 19% of the early adults reached the level of lower vocational education, 25% higher vocational education, 37.2% University of Applied Sciences and 18.8% reached university.

Table 4

**Table 4:** Descriptive statistics for incomplete and multiply imputed sample

	Incomplete sample				Multiply imputed sample			
Variables	Mean (SD)	Min	Max	*N*	Mean (SD)	Min	Max	*N*
WISC score	97.19 (15.00)	45	149	2220	97.16 (15.00)	45	149	2229
Socioeconomic status	–0.05 (0.80)	–1.94	1.73	2187	–0.05 (0.80)	–1.94	1.73	2229
Parental acceptance	3.21 (0.50)	1.17	4.00	2206	3.21 (0.50)	1.17	4.00	2229
Parental rejection	1.48 (0.31)	1.00	3.47	2205	1.48 (0.31)	1.00	3.47	2229
Peer acceptance	0.29 (0.16)	0.00	0.80	1064	0.28 (0.16)	0.00	0.80	2229
Peer rejection	0.13 (0.13)	0.00	0.85	1064	0.13 (0.14)	0.00	0.85	2229

	Frequencies (%)				Frequencies (%)			
Gender	50.7% girls			2229	50.7% girls			2229
	49.3% boys				49.3% boys			
Educational attainment	9.6% Lower vocational education			1430	19.0% Lower vocational education			2229
	24.8% Higher vocational education				25.0% Higher vocational education			
	41.5% University of Applied Sciences				37.2% University of Applied Sciences			
	24.1% University				18.8% University			

## Appendix B. Results of Binary Logistic Regressions

Table 5

**Table 5:** Cumulative binary logistic regression on educational attainment (*N* = 2229)

	0 = Lower vocational education 1 = Higher vocational education, University of Applied Sciences or university			0 = Lower or higher vocational education 1 = University of Applied Sciences or university			0 = Lower or higher vocational education or University of Applied Sciences 1 = University		
Variables	*B* (SE)	OR	FMI	*B* (SE)	OR	FMI	*B* (SE)	OR	FMI
Gender (1 = boys)	–0.46 (0.18)*	0.63	0.47	–0.14 (0.13)	0.87	0.29	–0.27 (0.15)	0.77	0.18
WISC score	0.07 (0.01)***	1.07	0.37	0.07 (0.01)***	1.07	0.27	0.07 (0.01)***	1.08	0.12
Socioeconomic status	1.01 (0.11)***	2.75	0.29	1.01 (0.09)***	2.76	0.24	1.18 (0.12)***	3.24	0.28
Parental acceptance	0.47 (0.17)**	1.63	0.36	0.48 (0.13)***	1.61	0.24	0.44 (0.17)**	1.55	0.14
Parental rejection	–0.01 (0.29)	0.99	0.41	–0.24 (0.22)	0.79	0.31	–0.65 (0.30)*	0.52	0.22
Peer acceptance	0.78 (0.81)	2.18	0.67	0.32 (0.64)	1.37	0.67	–0.55 (0.72)	0.58	0.55
Peer rejection	–1.17 (0.92)	0.31	0.72	–1.12 (0.65)	0.33	0.58	–1.82 (0.93)*	0.16	0.55
Parental × Peer acceptance	–0.18 (1.29)	0.84	0.50	0.34 (1.05)	1.41	0.43	–0.04 (1.34)	0.96	0.33
Parental × Peer rejection	0.06 (2.01)	1.06	0.56	1.10 (1.67)	3.02	0.42	3.07 (2.51)	21.62	0.43
Parental acceptance × Peer rejection	0.80 (1.37)	2.23	0.52	0.76 (1.30)	2.14	0.52	–0.36 (1.71)	0.70	0.34
Parental rejection × Peer acceptance	1.11 (2.11)	0.60	3.03	0.58 (1.81)	1.79	0.51	1.92 (2.39)	6.82	0.40
AIC	1576.3			2235.0			1539.5		
McFadden’s pseudo-*R*^2^	28.3%			27.7%			29.6%		
Nagelkerke pseudo-*R*^2^	38.7%			42.3%			42.0%		

**p* ≤ 0.05, ***p* ≤ 0.01, ****p* ≤ 0.001
